# Predictors of death at home among cancer patients in Israel: a population-based study

**DOI:** 10.1186/s12939-021-01437-y

**Published:** 2021-04-10

**Authors:** Yakir Rottenberg, Gil Goldzweig, Adir Shaulov

**Affiliations:** 1 Department of Oncology, Hadassah Medical Organization and Faculty of Medicine, Hebrew University of Jerusalem, Jerusalem, Israel; 2 School of Behavioral Sciences, The Academic College of Tel Aviv-Yafo, Rabenu Yeruham St, 86162 Yaffo, Israel; 3 Department of Hematology, Hadassah Medical Organization and Faculty of Medicine, Hebrew University of Jerusalem, Jerusalem, Israel

**Keywords:** Dying at home, Israel, Socioeconomic status, Cancer

## Abstract

**Background:**

Most cancer patients prefer to die at home; however, many die in hospital. The aim of the current study is to elucidate the association between dying at home and various personal factors in the Israeli population of cancer patients.

**Methods:**

Data on cancer incidence (2008–2015) and death (2008–2017) was provided by the Israeli Central Bureau of Statistics and the Israel National Cancer Registry. Binary logistic regression analyses were performed to assess odds ratios for death at home following cancer diagnosis while controlling for age, sex, ethnicity, years of education, residential socioeconomic score, and time from diagnosis. We also assessed the relation between place of death and specific cancer sites, as well as the time trend from 2008 to 2017.

**Results:**

About one quarter (26.7%) of the study population died at home. Death at home was most frequent among patients diagnosed with brain tumors (37.0%), while it was the lowest among patients with hematologic malignancies (lymphoma and leukemia, 20.3 and 20.0%, respectively). Rates of dying at home among patients with residential socioeconomic scores of 1, 2–9, and 10 were about 15, 30, and 42.9%, respectively. In patients from the 4th to the 7th decades of life, rates of death at home increased at a linear rate that increased exponentially from the 8th decade onwards. After controlling for potential confounders, predictive variables for death at home included age (OR = 1.020 per year, 95% CI 1.017–1.024), male sex (OR = 1.18, 95% CI 1.077–1.294), years of education (OR = 1.029 per year, 95% CI 1.018–1.040), and time from diagnosis (OR = 1.003 per month, 95% CI 1.001–1.005 all *p* < 0.001). No trend was seen from 2008 to 2013, while from 2014 to 2017 a slight increase in the rate of death at home was seen each year.

**Conclusions:**

These results indicate wide variability in death at home exists among patients of different ages, sex, education, socioeconomic status and time from diagnosis. These findings stress the importance of delivering quality palliative care at home, mainly for patients with hematologic malignancies, younger patients, and patients of very low socioeconomic status. Understanding the complex mechanisms whereby patient preferences and the above variables may determine the preferred place of death remains an important research priority.

## Background

Most cancer patients prefer to die at home, and death at home has emerged as an indicator of high-quality end-of-life care [1]. However, there is a gap between patients’ preferences and reality, and many if not most cancer patients die in hospital [2]. Potential barriers to dying at home include environmental (i.e., social support, healthcare availability, macrosocial factors), individual (i.e., age, sex, education, marital status, ethnicity, socioeconomic status, preference), and illness-related variables (i.e., disease type). Of these, environmental factors have been found to have the greatest impact on the rate of dying at home [3, 4].

Israel has universal and geographically distributed access to health care. Therefore, lack of healthcare availability may not present a major barrier to dying at home. In addition, the 1994 National Health Insurance Law provided a universal system of healthcare based on principles of justice, equality, and mutual assistance [5].

Currently, Israel has a total of 36 inpatient hospice beds for a population of nine million. Equitable, government mandated home hospice services of varying quality are available for all through the four health funds which deliver services to all Israeli citizens [6]. However, palliative and hospice services are not under strict governmental oversight as to when, how, and to what extent health providers are required to provide these services [5].

Statistics on place of death are not routinely reported in Israel, and information on the percentage of deaths occurring at home are not known. In this study we report, for the first time, on the place of death for Israeli cancer patients from 2008 to 2017, as well as the relationships between various individual factors and the probability of death at home for these patients. Defining specific populations in which death at home is achievable, as well as barriers to death at home, may provide helpful clues for potential interventions to promote death at home and higher quality end-of-life care.

## Material and methods

### Study population

This study was designed as a historical prospective cohort study. Cohort inception and baseline measurements were acquired from the Israeli Central Bureau of Statistics 1995 census. The target sample included a representative sample of the entire population that completed a comprehensive survey; 20% of the population in Israel aged 15 years and more, which were randomly sampled according to geographic residence. The file is constructed according to the census based on the long questionnaire encompassing 1,113,420 individuals.

### Cancer incidence

Data on cancer incidence was provided by the Israel National Cancer Registry, updated to 2010. The registry was established in 1960, and since 1982 it has received, by law, compulsory notifications of cancer incidence. Notifications include data from numerous sources including pathology reports, discharge summaries, and death certificates. The registry was found to be 95% complete for solid tumors [7]. All patients diagnosed with cancer from January 2008 to December 2015 were included in the current study. Among the study population, 33,428 cancer cases were diagnosed from 2008 to 2015. During the study period, 12,638 (37.8%) patients died of cancer. After excluding patients with missing data (place of death and missing dependent variables), the study included 9114 patients.

### Study variables

Variables assessed in relation to place of death included age at the time of cancer diagnosis, sex, ethnicity (Jewish vs. non-Jewish), years of education (continuous variable), time from cancer diagnosis until death and residential socioeconomic score (RSS). RSS is an ordinal variable based on weighting of 14 variables that measure the social and economic level in four domains – demographics, education, standard of living, and employment. The weighting creates a national metric by which neighborhoods or localities are divided into residential socioeconomic clusters, with 1 being the lowest cluster, and 10 being the highest. We also assessed the relation between place of death and specific cancer types (in each category above 100 confirmed deaths), as well as the time trend from 2008 to 2017.

### Survival outcome

Place of death was determined using the Israel Population Registry, Central Bureau of Statistics cause of death file, which was current to December 31st, 2017. Mortality data are considered to be 100% complete for all individuals dying in Israel.

### Statistical analyses

Descriptive statistics was used in order to describe the distribution of the study variables in both groups (death at home vs. death at hospital). Continuous variables (age, years of education, and time from diagnosis) were compared between the death at home and death at hospital groups using the Student’s t-test. Categorial (sex, ethnicity) and ordinal variable (RSS) were compared using the χ2 test. A binary logistic regression analysis was constructed in order to examine the unique contribution of each variable over and above others. Predictors included age, sex, ethnicity, education, residential socio-economic position, and time from diagnosis until death. The study proposed the following logistic regression model: Y = β + Βx1 + βx2 + βx3 + Βx4 + Βx5+ Βx6 + ε” (Where Y = Die at home/ Hospital; X1 = Age; X2 = Male = 1; X3 = Jewish = 1; X4 = RSS; X5 = Education years; X6 = months from cancer diagnosis; ε is the error term. All statistical tests were two tailed with *P* < 0.05 considered statistically significant. The SPSS program was used for the statistical analysis.

This study was approved by both the ethics and confidentiality committees of the Israeli Central Bureau of Statistics and the Health Ministry Director.

## Results

Death at home (Table 1) was most frequent among patients diagnosed with brain tumors (37.0%), while it was lowest among patients with hematologic malignancies (lymphoma and leukemia, 20.3 and 20.0%, respectively).

**Table 1:** Baseline characteristics of the study population

Variable	Hospital (***N*** = 6479)	Home (***N*** = 2635)	***P*** value
**Age at diagnosis (years)** mean ± SD	70.9 ± 13.2	74.0 ± 13.0	< 0.001
**Sex (male)** Frequency (percent)	3113 (48.0%)	1383 (52.5%)	< 0.001
**Ethnicity (Jewish)** Frequency (percent)	5617 (86.7%)	2378 (90.2%)	< 0.001
**RSS**^**a**^ mean ± SD	6.0 ± 2.0	6.2 ± 2.0	< 0.001
**Education (years)** mean ± SD	10.2 ± 4.6	10.7 ± 4.5	< 0.001
**Time from cancer diagnosis (months)** mean ± SD	23.2 ± 25.5	24.7 ± 26.0	< 0.01
**Cancer type**			
**Brain (*n*** **= 411)**	259 (63.0%)	152 (37.0%)	
**Pancreas (*n*** **= 666)**	427 (64.1%)	239 (35.9%)	
**Uterus (*n*** **= 490)**	124 (64.2%)	69 (35.8%)	
**Melanoma (*n*** **= 344)**	221 (64.2%)	123 (35.8%)	
**Prostate (*n*** **= 512)**	336 (65.6%)	176 (34.4%)	
**Colorectal (*n*** **= 1352)**	802 (66.7%)	450 (33.3%)	
**Renal (*n*** **= 216)**	149 (69.0%)	67 (31.0%)	
**Breast (*n*** **= 840)**	595 (70.8%)	245 (29.2%)	
**Ovary (*n*** **= 230)**	162 (70.4%)	68 (29.6%)	
**Stomach (*n*** **= 458)**	324 (70.7%)	134 (29.3%)	
**Lung (*n*** **= 1428)**	1074 (73.3%)	381 (26.7%)	
**Bladder (n = 490)**	369 (75.3%)	121 (24.7%)	
**Liver (*n*** **= 170)**	129 (75.9%)	41 (24.1%)	
**Lymphoma (*n*** **= 316)**	252 (79.7%)	64 (20.3%)	
**Leukemia (*n*** **= 795)**	636 (80.0%)	159 (20.0%)	

^a^*RSS* Residential Socioeconomic Score

**Fig. 1:**
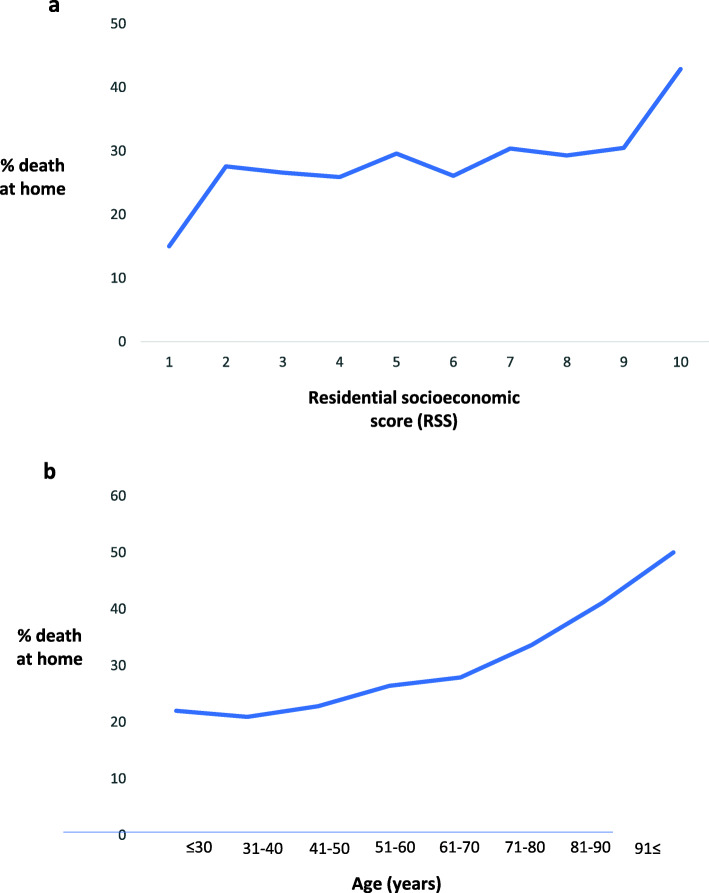
Rates of death at home according by RSS and age group

A comparison between rates of death at home in all RSS and age groups is presented in Fig. 1. Thirty percent of patients with RSSs of 2–9 died at home. In contrast, among the highest RSS, 42.9% of cancer patients died at home, while only 15.0% among the lowest RSS died at home. Among various age groups, death at home occurred in 20.9% of those aged 31–40, 22.8% in those aged 41–50, 26.4% in those aged 51–60, 27.9% in the those aged 61–70, 33.6% in those aged 71–80, 41.1% in those aged 81–90, and 50% in those aged 91 years and above.

**Fig. 2:**
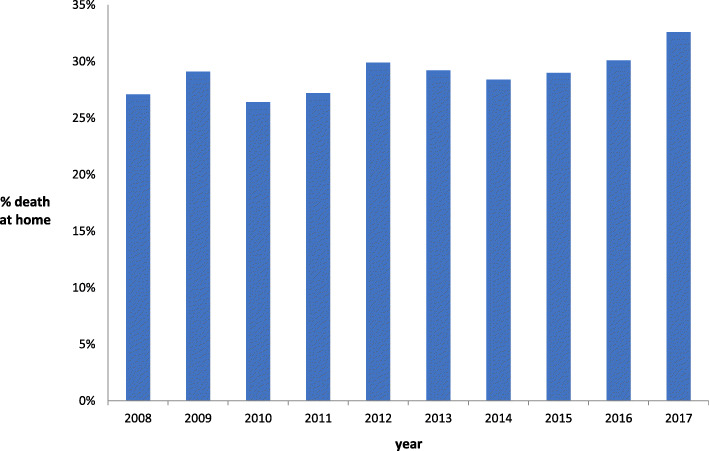
Rates of death at home between the years 2008 and 2017

During the study period (2008–2017), rates of death at home ranged from 27.1% (2008) to 32.6% (2017). No trend was seen from 2008 to 2013, while from 2014 to 2017 a slight increase in death at home was seen each year (Fig. 2).

Death at home was associated with higher age at diagnosis (74.0 ± 13.0 years vs. 70.9 ± 13.2, *p* < 0.001), male sex (52.5% vs. 48.0%, *p* < 0.001), Jewish ethnicity (90.2% vs. 86.7%, *p* < 0.001), higher RSS (6.2 ± 2.0 vs. 6.0 ± 2.0, *p* < 0.001), years of education (10.7 ± 4.5 years vs. 10.2 ± 4.6 years, *p* < 0.001), and months from diagnosis (24.7 ± 26.0 months vs. 23.2 ± 25.5, *p* < 0.01). In binary logistic regression analysis, predictive variables for death at home included age (OR = 1.020 per year, 95% CI: 1.017–1.024), male sex (OR = 1.18, 95% CI: 1.08–1.29), years of education (OR = 1.03 per year, 95% CI: 1.02–1.04), and time from diagnosis (OR = 1.002 per month, 95% CI: 1.000–1.004). On the other hand, ethnicity (OR = 1.12, 95% CI: 10.95–1.33) and RSS (OR = 1.003, 95% CI: 0.977–1.029) were not associated with death at home (Table 2). The predicted variable was place of death. This model is reflected in the following equation: ln (Odds (Die to home/Die Hospital)) = (− 2.936) + ln (1.020)*Age + ln (1.180)*[Male = 1] + ln (1.124)*[Jewish = 1] + ln (1.003)*RSS + ln (1.029)*Education_years + ln (1.003)*time_from_dx_months. The multivariate logistic regressionwith all variables had indices pseudo-R2 Cox and Snell = 0.018 and Nagelkerke = 0.026, which calculate the log likelihood for the model compared to the log likelihood for a baseline model, scaled the full range from 0 to 1 with the largest value being “best” according to this measure.

**Table 2:** A binary logistic regression for death at home

Variable	Odds Ratio	95% Confidence Interval (CI)	***P*** value
**Age at diagnosis (years)**	1.020	1.017–1.024	< 0.001
**Sex (male)**	1.180	1.077–1.294	< 0.001
**Ethnicity (Jewish)**	1.124	0.953–1.325	0.166 NS^b^
**RSS**^**a**^	1.003	0.977–1.029	0.849 NS^b^
**Education (years)**	1.029	1.018–1.040	< 0.001
**Time from cancer diagnosis (months)**	1.003	1.001–1.005	< 0.001

^a^*RSS* Residential Socioeconomic Score

^b^*NS* Not significant

## Discussion

In this large, Israeli, population-based study, we found an association between place of death and age, male sex, years of education, and time from diagnosis. Rates of death at home were constant among most socioeconomic residential scores (~ 30% for levels 2–9). Rates of death at home differed only between the highest and the lowest RSS (42.9% deaths at home at level 1 and 15.0% at level 10). Death at home rates were highly variable among the different cancers: the highest rate was seen among patients with brain tumors (37.0%), while the lowest rate was among patients with hematologic malignancies (lymphoma and leukemia, 20.3 and 20.0%, respectively). In addition, a slight increase was seen each year during the last years of the study.

The association found between dying at home and socioeconomic status has been previously reported in other countries [8]. The mechanisms whereby socioeconomic status affects one’s ability to die at home may involve various factors: whether a person lives alone, the ability to provide adequate care at home, adequate access to care services, care-related costs, control over monetary resources, the ability to secure local support, and personal preferences [9]. Socioeconomic status may, however, influence or be influenced by other dominant variables. For example, higher socioeconomic status may contribute to a more stable home environment and increased patient comfort with home death, and more resources may be available to pay for formal caregivers and strong support networks, thus increasing the likelihood of dying at home. Rather than a direct effect on the likelihood of dying at home, higher socioeconomic status may be the result of higher education, which in itself may result in a higher likelihood of dying at home [10]. High socioeconomic status is also characteristic of those seeking physician-assisted suicide as a tool to achieve the sense of control that is of importance to this population [11, 12]. Dying at home may present a similar measure of control over the uncontrollable circumstances of approaching end of life. On the other hand, the association between high socioeconomic status and death at home may reflect unequal opportunities for optimal delivery of care according to patients’ wishes at end of life.

The constant rate of dying at home among interim levels of socioeconomic status supports the importance of defining socioeconomic status as an ordinal variable rather than a continuous variable when attempting to understand complex mechanisms whereby socioeconomic status affects one’s ability to die at home. The regression included all the RSS levels and since home death rates were almost constant across subsequent levels the differences between the extreme levels were not identified by the model. The fact that for most RSS levels in Israel home death rates were constant may have multiple explanations. Israel has National Health Insurance coverage and highly accessible health services [13], which may decrease the impact of socioeconomic status on accessibility to health care, resulting in differences only between extreme levels of RSS. In addition, the relationship between low socioeconomic status and dying in hospital may be attenuated in Israel by the nature of the population in areas with low RSS. In these areas, there are more ultra-orthodox Jews and Muslim Arabs. These populations are characterized by a preference to die at home, as well as strong social support that may help fulfill the wish to die at home [14–17]. Yet, the smallest rates of dying at home among patients at the lowest RSS may reflect inability to provide adequate care at home due to high indirect care-related costs and incapacity for delivery optimal care due to limited size apartment. Thus, the current results highlight the need for improvement of end-of-life care at home mainly among patients in the lowest RSS.

In our study, while death at home occurred at a relatively fixed rate in patients from 31 (20.9%) to 70 (27.9%) years of age, the oldest patients exhibited a significant increase in the percentage of those dying at home: 33.6% among those aged 71–80, 41.1% among those aged 81–90, and reaching 50% in those 91 years old and above. A similar association between age and dying at home has been reported in other countries, primarily in Singapore [18]. However, in Scotland, age has been found to be inversely associated with dying at home [18, 19]. This variability likely reflects cultural differences in the care of the elderly as well as availability of personal care workers. The strong association found between age and death at home of patients over 81 years old in our study is remarkable. These results may be related to the fact that the oldest old cancer patients in Israel invest resources in close relations and attribute fundamental importance to family caregivers, turning their thoughts to their surroundings, relatives, and family [20], and they may therefore prefer death at home in their accustomed environment [21]. Dying at home is fraught with illness and caregiver-related uncertainty [22]. Illness-related uncertainty may be less prevalent for the oldest patients, and indeed many of them may already have formal caregivers, thus reducing caregiver-related uncertainty.

The lowest rates of death at home among patients with hematologic malignancies may reflect a younger sub-population. On the other hand, end-of-life care for patients with hematologic malignancies deficient compared to treatment for other solid tumors [23]. More aggressive treatments at the end of life resulting from the continuing discovery of cures for advanced diseases, as well as the, often rapid, pace of decline near death, have been reported among patients with hematologic malignancies [23]. Another barrier to efficient end-of-life care for this population is unrealistic expectations from both physicians and patients. Also, quality measures developed for solid tumors (such as blood transfusions) may be unacceptable for patients with blood cancers [23].

In contrast to those suffering from hematologic malignancies, patients with brain tumors have the highest rates of death at home. Swallowing difficulties combined with decreased function and consciousness are frequently seen among these patients during the end-of-life period [24]. These symptoms frequently appear a few weeks before death. During this period, major challenges are to efficiently provide basic adult daily activities and to deal with minor medical problems, which can frequently be done at home.

Rates of death at home gradually increased during the years 2014–2017. We hypothesize that these changes are the result of the National Program for Palliative Patients, launched in 2015.

### Strenghts and limitations

The strengths of this study include the use of large national databases which allowed us to adjust the results for potential confounders and to mitigate selection and information biases. However, this study did not include information on patients’ or family members’ preferences for place of death, both of which have a great impact on the place of death [8, 25].

## Conclusions

Relatively little emphasis has been placed on various factors and their relation to place of death among cancer patients in Israel, although previous studies have reported that home is the preferred place of death and an indicator of high-quality end-of-life care [1, 2]. This study is, to our knowledge, the first large population-based report on factors associated with dying at home among cancer patients in Israel. Our data support an association between age, sex, years of education, time from diagnosis, and dying at home. Further analysis shows the lowest rates of death at home are found among patients with hematologic malignancies, while a slight increase in rates of death at home in the entire study population was reported each year during the last years of the study. Our data add to other arguments supporting the increased need for access to palliative care resources for patients with hematologic malignancies. This data should also help open the door for research into the causes of reduced death at home for younger patients and patients of very low socioeconomic status. Further studies should also investigate the association between patient preferences and actual place of death, as well as the complex mechanisms whereby these variables are associated with death at home. In addition, continued monitoring of death at home rates among this population over the following years is needed in order to confirm the observed trend of an increase in home death over the last years of this study. These data will enable the development of focused intervention programs for specific sub-populations.

## Data Availability

The datasets generated and/or analyzed during the current study are not publicly available due to the obligation regarding safeguard the confidentiality of the data by the Israeli Central Bureau of Statistics, but are available from the corresponding author upon reasonable request.

## Abbreviation

RSS: Residential socioeconomic score
